# Epstein-Barr virus IL-10 gene expression by a recombinant murine gammaherpesvirus *in vivo* enhances acute pathogenicity but does not affect latency or reactivation

**DOI:** 10.1186/2042-4280-5-1

**Published:** 2014-09-24

**Authors:** Gary J Lindquester, Kimberly A Greer, James P Stewart, Jeffery T Sample

**Affiliations:** 1 Department of Biology, Rhodes College, Memphis, TN 38112, USA; 2 Department of Infection Biology, Institute of Infection and Global Health, University of Liverpool, Liverpool L3 5RF, UK; 3 Department of Biochemistry, St. Jude Children’s Research Hospital, Memphis, TN 38105, USA; 4 Current Address: Department of Microbiology and Immunology, The Pennsylvania State University College of Medicine, Hershey, PA 17033, USA

**Keywords:** Gammaherpesvirus, Epstein-Barr virus, IL-10, Interleukin 10, Immune modulation

## Abstract

**Background:**

Many viral genes affect cytokine function within infected hosts, with interleukin 10 (IL-10) as a commonly targeted mediator. Epstein-Barr virus (EBV) encodes an IL-10 homologue (vIL-10) expressed during productive (lytic) infection and induces expression of cellular IL-10 (cIL-10) during latency. This study explored the role of vIL-10 in a murine gammaherpesvirus (MHV) model of viral infection.

**Methods:**

The EBV vIL-10 gene was inserted into MHV-76, a strain which lacks the ability to induce cIL-10, by recombination in transfected mouse cells. Mice were infected intranasally with the recombinant, vIL-10-containing MHV-76 or control virus strains and assayed at various days post infection for lung virus titer, spleen cell number, percentage of latently infected spleen cells and ability to reactivate virus from spleen cells.

**Results:**

Recombinant murine gammaherpesvirus expressing EBV vIL-10 rose to significantly higher titers in lungs and promoted an increase in spleen cell number in infected mice in comparison to MHV strains lacking the vIL-10 gene. However, vIL-10 expression did not alter the quantity of latent virus in the spleen or its ability to reactivate.

**Conclusions:**

In this mouse model of gammaherpesvirus infection, EBV vIL-10 appears to influence acute-phase pathogenicity. Given that EBV and MHV wild-type strains contain other genes that induce cIL-10 expression in latency (e.g. LMP-1 and M2, respectively), vIL-10 may have evolved to serve the specific role in acute infection of enlarging the permissive host cell population, perhaps to facilitate initial survival and dissemination of viral-infected cells.

## Background

Viruses from a range of virus families exert direct influence on host cytokine responses. Such influence can be mediated through expression of viral homologs of host cytokines or cytokine receptors, or through expression of viral factors that alter expression of host cytokines [1-3]. Several herpesviruses such as Epstein-Barr virus (EBV) [4], equid herpesvirus 2 [5], ovine herpesvirus 2 [6], and primate cytomegaloviruses [7-9] encode a homolog to the IL-10 gene. Infection of B cells by EBV results in expression of virus-encoded IL-10 (vIL-10, encoded by the *BCRF1* gene) as well as induction of the endogenous cellular IL-10 (cIL-10) gene. vIL10 mRNA is detectable within six hours of EBV infection *in vitro*[10] and thus is expressed early in infection [11]. In fact, EBV virions carry a number of viral mRNAs including those encoding vIL-10, which may be translated immediately upon infection [12]. Subsequently, expression of the cIL-10 gene is induced twenty to forty hours post-EBV infection [10,13,14] and is upregulated by the EBV latency-associated LMP-1 and small non-coding RNAs (EBERs) [15,16]. The differential timing of expression of these two IL-10 forms may reflect differences in their respective roles in viral infection.

IL-10 is a highly pleotropic, regulatory cytokine with differential effects in T-cell populations that generally reduce inflammation and cytotoxic responses, while favoring a humoral immune response [17]. The EBV vIL-10 [4], like its cellular counterpart, inhibits cytokine synthesis [18]. EBV vIL-10 is 84% homologous to human IL-10, with most divergence occurring at the N terminus [4] resulting in an altered N-terminal structure [19]. cIL-10 and vIL-10 enhance B-cell viability, whereas only cIL-10 upregulates MHC II on B cells [20]. vIL-10 also lacks cIL-10’s ability to stimulate mast cells [21] and to induce proliferation of mature and immature thymocytes [22]. EBV vIL-10 has 1000-fold lower affinity for the IL-10 receptor, perhaps explaining its greatly reduced ability to inhibit IL-2 production by helper T cells [23]. Thus, the viral and cellular homologs share many immunosuppressive activities, while vIL-10 generally lacks cIL-10’s immunostimulatory functions. These differences are attributed primarily to a single amino acid substitution [24].

In earlier studies involving an EBV mutant in which the vIL-10-encoding gene was deleted, vIL-10 was concluded to have no effect on replication, immortalization and establishment of latency within B cells *in vitro*, and to have no effect on tumorigenicity when the resulting EBV-infected B lymphoblastoid cell lines were injected into SCID mice [11]. More recent *in vitro* studies with vIL-10-deficient EBV virus have demonstrated that vIL-10’s early expression protects infected B cells by altering the cytokine response, reducing NK cell killing, and inhibiting CD4+ T cell activity [12]. *In vivo*, expression of the vIL-10 gene of the betaherpesvirus rhesus cytomegalovirus (RhCMV), significantly limits innate immune responses to primary infection, which in turn reduces both T- and B-cell responses [25]. However, the applicability of the latter study to understanding EBV vIL-10 function is questionable given the low (27%) homology of RhCMV vIL-10 with cIL-10 [9] and the high binding affinity of RhCMV vIL-10 relative to EBV vIL-10 for the IL-10 receptor [26]. To date, studies probing the nuances of EBV vIL-10 in gammaherpesvirus infection have not been presented in an *in vivo* model of viral pathogenicity.

Murine gammaherpesvirus 68 (MHV-68, γHV68, murid herpesvirus 4) infection of laboratory mice serves as a tractable animal model for gammaherpesvirus pathogenesis [27]. Upon intranasal (i.n.) inoculation of mice, MHV-68 rapidly establishes an acute, productive infection of alveolar epithelial cells which is essentially cleared about 10 days post-infection (p.i.) [28]. As the acute phase resolves, a syndrome similar to EBV-induced infectious mononucleosis ensues. This phase is characterized by splenomegaly [29], non-antigen-specific B-cell activation [30], and peripheral blood lymphocytosis primarily reflecting the expansion of CD8+ T cells expressing a Vβ4 T-cell receptor [31]. This syndrome peaks at day 14 p.i. and resolves by about day 21 p.i. Latent virus has been detected in peritoneal macrophages [32], splenic macrophages and dendritic cells [33], and B cells [33-36]. B cells are likely to be the means for trafficking MHV-68 from the lung to the spleen [37] and expression of vtRNAs, a marker for latency, has been localized to the germinal centers in the spleen [38-40]. CD4+ T cells are required for MHV-68-induced splenomegaly [29,41], while CD8+ T cells are critical for limiting productive pulmonary infection and for the resolution of splenomegaly [35,41-43].

MHV-76, a variant of MHV-68, contains a deletion of 9538 bp within the left end of the unique-sequence domain of the MHV-68 prototype genome – the region which includes the MHV-68 genes *M1*-*M4* as well as eight vtRNA genes [44,45]. In comparison to MHV-68, MHV-76 is cleared more rapidly from the lungs and induces less pronounced splenomegaly and fewer numbers of latently infected cells in the spleen, although replication of the viruses in culture does not differ [44,45]. The *M2* gene encodes a latency-associated protein that serves as a target for cell-mediated immunity [46]. The M2 protein binds Vav signaling proteins and promotes cell proliferation and survival [47]. MHV-68 strains lacking only M2 expression show the same reduction in latency and reactivation as MHV-76; however, they do not exhibit MHV-76’s reduction of splenomegaly [48-50]. While MHV-68 does not carry a native vIL-10 gene, M2 stimulates cIL-10 expression [51,52]. It has been proposed that M2’s role in stimulating cIL-10 and its resulting effects may represent a conserved gammaherpesvirus strategy that is also represented by the EBV vIL-10 gene [51], although, as noted above, the timing of vIL-10 expression differs from that of cIL-10 induction. Therefore, we sought to determine the effects of vIL-10 expression on viral pathogenesis in a murine gammaherpesvirus strain lacking *M2* (MHV-76). Here we show that vIL-10 expression by MHV-76 enhances spleen cell proliferation and viral titers in the lung during acute infection, but does not affect splenic latency or reactivation of virus replication from latently infected cells.

## Methods

### Cell and virus culture

All MHV strains were propagated in NIH-3T3 (ATCC) cells in DMEM (GIBCO) supplemented with 10% fetal bovine serum (HyClone), penicillin (100U/ml) and streptomycin (100 μg/ml) (GIBCO) at 37°C in a humidified 5% CO_2_ atmosphere essentially as described [28]. Viral titers were determined in a serial dilution plaque assay by fixing [10% formalin (Fisher)] and staining [0.1% toluidine blue (LabChem)] 3–5 days p.i.

### Generation of recombinant viruses

Promoter: Pgp150, the MHV-68 late gene promoter for the M7 gene (expressing gp150), was isolated from MHV-68 DNA by PCR amplification of a 660 base pair (bp) fragment that begins 3 bp upstream of the M7 ORF start codon and extends upstream. The Pgp150 primers (5′-GAGTAGATCTTAAGGGAGAGCGATGAGG-3′ and 5′-CAGTAAGCTTGAGGGTTTTATAGCGTCAC-3′) included *Bgl*II and *Hind*III restriction enzyme sites at the upstream and downstream ends, respectively. Promoters were inserted into the *Bgl*II and *Hind*III sites of the pGL3-Basic (sans promoter) luciferase reporter plasmid (Promega). Luciferase expression assayed with the Dual-Luciferase system (Promega) confirmed the activity of the promoters in NIH-3T3 cells transfected (FuGene, Roche) with the resulting plasmids.

vIL-10: The vIL-10 gene (encoded by the EBV *BCRF1* ORF) was amplified by PCR from EBV (Akata strain) DNA using primers that generated *Nco*I and *Xba*I restriction sites at the upstream and downstream ends, respectively. Primers were 5′-GTGACCATGGAGCGAAGGTTAGTG-3′ and 5′-AGTGTCTAGATGCACCCATCTCCTGCTTC-3′. The amplified product was cloned into the Pgp150-containing plasmid in place of the luciferase gene to create the vIL-10 expression cassette. Positive ELISA (Pierce) confirmed vIL-10 expression from plasmids in transfected NIH-3T3 cells.

**Figure 1:**
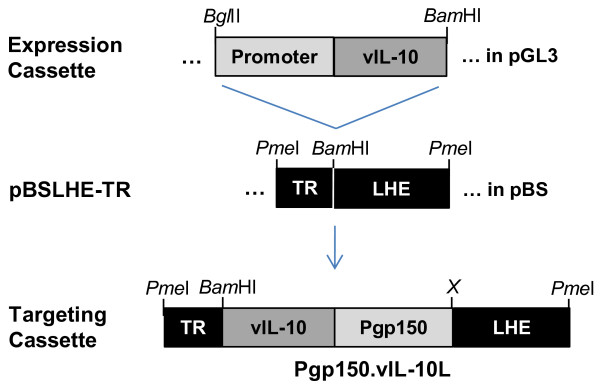
**Schematic of approach to generating recombinant viruses.** Expression cassettes were created in the pGL3-Basic vector (Promega) by insertion of the EBV vIL-10 gene along with the MHV76 gp150 promoter (Pgp150). Targeting cassettes were created by insertion of the expression cassette between the MHV76 terminal repeat segment (TR) and unique sequences of the left hand end (LHE, prototype orientation) in the pBSLHE-TR vector (see Methods). The targeting cassette was generated for this study with transcription from Pgp150 in the leftward direction, as determined by the asymmetric regeneration of the *Bam*HI site during insertion (X = no restriction site). The targeting cassette and MHV76 DNA were co-transfected into NIH-3T3 cells for recombination. Once isolated, recombinant viral DNA was co-transfected along with the TR-LHE segment from pBSLHE-TR into NIH-3T3 cells to generate revertant viruses.

Targeting cassette: Plasmid pBS76LHE (courtesy of James Stewart) contains an approximately 3-kbp fragment from the left hand end (LHE) unique sequence of the MHV-76 genome [53] and was modified to generate pBS76LHE-TR as follows. A portion of a terminal repeat fragment and its immediately adjacent unique sequence was amplified from MHV-68 DNA by PCR under conditions favorable for GC rich sequences (Roche). Primers were 5′-AGGCAGGCACCAACAG-3′ and 5′-CAGCATCAGCCCCGGATCTC-3′. This fragment, designated TR, represents terminal repeat sequences immediately to the left (in the prototype orientation) of the LHE fragment in MHV-76. TR was inserted next to the LHE fragment in pBS76LHE to generate pBSLHE-TR; a *Bam*HI site separates TR from LHE. Furthermore, *Pme*I restriction enzyme sites were inserted on either side of the TR-LHE sequence to be able to liberate the TR-LHE targeting cassette. Next, the vIL10 expression cassette was liberated from its plasmid as a fragment with a *Bgl*II restriction site on the upstream end and a *Bam*HI restriction site on the downstream end. This fragment was inserted into the *Bam*HI site of pBS76LHE-TR and restriction enzyme analysis revealed clones containing the expression cassette in either orientation bounded by the TR and LHE components. Figure 1 shows a general schematic of construction of the targeting cassette.

Recombination and purification: NIH-3T3 cells grown in 6-well plates were co-transfected using FuGene (Roche) with MHV-76 DNA (1 μg, isolated essentially as described [54]) and targeting cassette (2–3 μg of *Pme*I-digested targeting-construct plasmid). Following the development of plaques, cultures were harvested and subjected to three rapid freeze/thaw cycles to release cell-associated virus. Stocks were serially diluted to infect NIH-3 T3 cells in 96-well plates. DNA was isolated from wells developing single plaques by QiaAmp (Qiagen) and screened by PCR for the presence of the vIL-10 gene. This limiting-dilution screening was repeated for five or six rounds until all plaques were PCR positive for vIL-10. Positive ELISA (Pierce) confirmed vIL-10 expression from recombinant viruses in infected NIH-3T3 cells. Revertant control viruses were generated using the preceding procedures by co-transfection of cell cultures with recombinant virus DNA and the TR-LHE fragment lacking an expression cassette insert. All recombinant DNA work was conducted under protocols approved by the Institutional Biosafety Committee following US federal guidelines.

### *In vitro* growth curves

NIH-3T3 cell cultures (70-80% confluent) were infected at a multiplicity of infection (MOI) of 5. Virus was allowed to adsorb for one hour at 37°C, and cells were washed three times with fresh medium to remove unbound virus. Samples were taken at time zero and appropriate time points thereafter by scraping the cells and collecting by aspiration. Cells were freeze-thawed three times to release cell-associated virus, and viral titers were determined by plaque assay.

### Inoculation and sampling of mice

Four- to six-week-old male BALB/c mice (Jackson Laboratories) under light anesthesia (isoflurane, 1.5-2.5%, by inhalation) were inoculated i.n. with 2 × 10^5^ pfu virus [45]. At various times p.i., mice were euthanized by CO_2_ asphyxiation followed by cervical dislocation. Lungs were removed and snap frozen. Spleens were removed and held briefly in tissue culture medium. After spleens were weighed, they were homogenized by passage through a mesh screen, and red blood cells were lysed in Red Blood Cell Lysing Buffer (Sigma-Aldrich). Leukocytes were recovered from the pellet following centrifugation by resuspension in tissue culture medium, and aliquots of cells were counted to calculate number of leukocytes per spleen. All animal work was conducted under protocols approved by the Institutional Animal Care and Use Committee following US federal guidelines.

### Quantification of lytic virus

Spleens and lungs were harvested from euthanized mice various days p.i. and homogenized (Mini-BeadBeater-8, as described [36]). Supernatants were freeze-thawed and clarified by centrifugation. Virus titers were determined by plaque assay of serial dilutions on NIH-3T3 cells.

### Quantification of latent virus

Viral DNA was detected using a limiting-dilution, nested PCR assay for the MHV-68 ORF50 gene with single-copy sensitivity essentially as described [32,50].

### Reactivation assay

The limiting-dilution assay as described by Weck [36] was used to assess reactivation from latently infected spleen cells. Briefly, mouse embryonic fibroblast (MEF) cells (ATCC), were plated in 96-well culture plates and inoculated with limiting-dilutions of splenic leukocytes or splenic-cell lysates, and results were analyzed after 14–21 days with subtraction of any detectable lytic virus present in the lysate. Statistical analysis of all experiments was conducted using GraphPad Prism software.

## Results

### Construction of recombinant MHV-76 and expression of vIL-10

**Figure 2:**
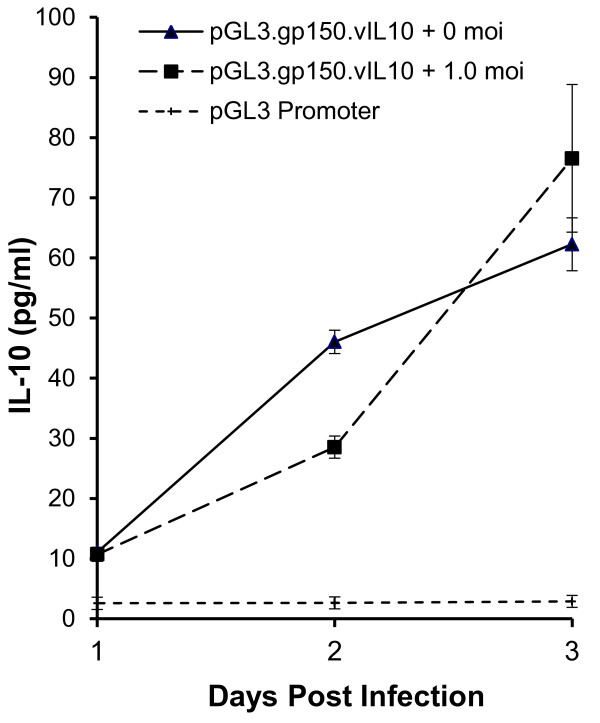
**vIL-10 is expressed in transfected cells.** NIH-3T3 cells were transfected with plasmid containing the Pgp150.vIL10 expression cassette or control plasmid (pGL3-Promoter) and infected or not with MHV-76 at a MOI of 1. After 24, 36, and 48 hours, supernatant was tested for the presence of vIL-10 by ELISA. Data points represent the averages of two readings of each sample in duplicate experiments. Error bars indicate +/- 1 standard deviation.

For expression of the EBV vIL-10 gene in MHV-76, we utilized the MHV-68 M7 late-gene promoter which allows transcription of the gp150 protein. A 660-bp fragment from upstream of the gp150 start codon was amplified by PCR and inserted directly upstream of the luciferase gene in Promega’s pGL3-basic vector. The promoter was successful in driving expression of luciferase when the plasmid was transfected into NIH-3T3 cells with or without MHV-76 viral infection (data not shown). The luciferase coding sequences were then replaced with the coding sequences for EBV vIL-10. The new constructs were successful in expressing vIL-10 in NIH-3T3 cells with similar expression levels in uninfected cells and in cells infected with MHV-76 (Figure 2).

**Figure 3:**
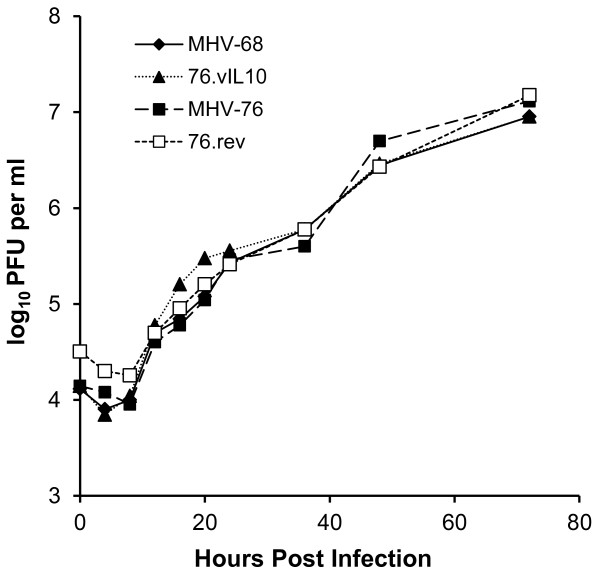
**Viruses have equivalent *in vitro *growth properties.** NIH-3T3 cells were infected at a MOI of 5. Cells were harvested at various times post-infection, and viral titers were determined in duplicate. Data points represent average titers at each sampling time.

The gp150-promoter/vIL-10 cassette was then inserted between the terminal repeat and the unique sequences of the left hand end of the cloned MHV-76 DNA to create the targeting cassette (Figure 1). The targeting cassette was liberated from its plasmid by restriction enzyme digestion and co-transfected with MHV-76 DNA into NIH-3T3 cells to allow for recombination within the TR and LHE sequences, effectively inserting the vIL-10 gene into the MHV-76 genome. Putative recombinant viruses (designated 76*.*vIL10) were screened by PCR upon multiple rounds of limiting-dilution infections *in vitro* until a purified culture of vIL-10 PCR-positive virus was obtained. Expression of vIL-10 by recombinant viruses was confirmed by ELISA, which demonstrated a mean concentration of vIL-10 three days p.i. in cell culture supernatant of 11 ng/ml (standard deviation = 5.5 ng/ml; three trials with duplicate samples per trial). Finally, recombinant virus was co-transfected with a fragment containing the TR-LHE contiguous sequence in order to create a revertant virus strain (designated 76.rev). Insertion and integrity of the promoter and gene sequences were confirmed by DNA sequence analysis and MHV-76, 76.vIL10, 76.rev, and MHV-68 virus strains all were shown to have very similar *in vitro* growth rates (Figure 3).

### vIL-10 enhances acute viral titers in lungs

**Figure 4:**
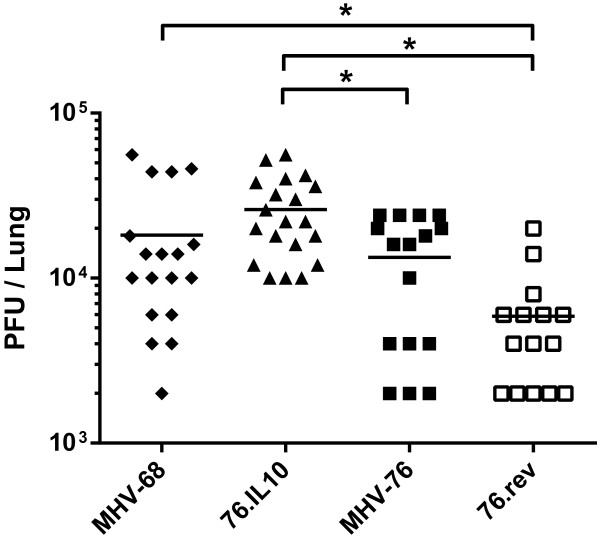
**vIL10 increases acute-phase titer in lungs.** Four- to six-week-old BALB/c mice were infected i.n. with 2x10^5^ pfu of virus in 40 milliliters. Viral titers from homogenized left lungs were determined on NIH-3T3 cells. Data represent three independent experiments with titers performed in duplicate. Pairwise statistical analysis shows no significant differences between titers of MHV68 or 76 or between either of these viruses and the recombinants. Statistical significance by unpaired t-test with Welch’s correction is indicated (*): 76.vIL10 vs MHV-76 (*P* = 0.0025), 76.vIL10 vs 76.rev (*P* < 0.0001), MHV-68 vs 76.rev (*P* = 0.0076).

Previous studies have shown that MHV-76 titer in infected-mouse lungs peaks at day 4 p.i., while MHV-68 titer peaks significantly higher and at day 6 p.i. [45]. In these experiments (Figure 4), viral titers in lungs at day 5 p.i. were significantly higher for the recombinant 76.vIL10 than either MHV-76 (*P* = 0.0025*)* or the revertant control strain, 76.rev (*P* < 0.0001). These results demonstrate that vIL-10 allows enhanced acute viral titers in lungs. Furthermore, no significant difference was seen in titers at this time point for MHV-68 versus 76.vIL10. While MHV-68 titers were significantly higher than those of 76.rev (*P* = 0.0076), the marginally higher mean titer of MHV-68 versus MHV-76 was not significant.

### vIL-10 promotes an increase in spleen cell number

**Figure 5:**
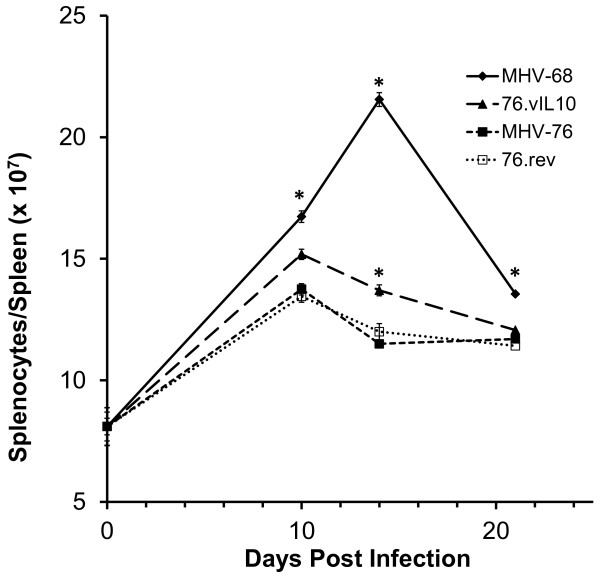
**vIL10 increases spleen cell count at day 14.** Mice were infected as indicated in Figure 4. Data represent four independent experiments with five mice per group. Spleens were harvested on days 10, 14, and 21 p.i. Spleens of five mice were pooled and disrupted, and spleen cells were counted. Spleen-cell-count data for Day 0 represent the average of five spleens from uninfected mice. Bars represent standard error of the mean. Statistical significance by unpaired t-test with Welch’s correction is indicated (*): MHV-68 vs MHV-76 (day 10, *P* = 0.0106; day 14, *P* < 0.0001; day 21, *P* = 0.0005) and 76.IL10 vs MHV-76 (day 14, *P* < 0.0001).

The development of splenomegaly, a characteristic of MHV acute phase infection that normally peaks from day 10–14 p.i., was assessed by determining the total number of leucocytes in infected-mouse spleens. Splenocyte counts were determined at days 10, 14, and 21 p.i. (Figure 5). As expected [45], MHV-68 compared to MHV-76 resulted in a significant increase in number of splenocytes throughout the measured course of infection, with peak expansion of the population at day 14 (day 10, *P* = 0.0106; day 14, *P* < 0.0001; day 21, *P* = 0.0005). Splenocyte counts peaked at day 10 p.i. in mice infected with either the vIL-10-containing strain 76.vIL10, or its parent strain, MHV-76. However, at day 14 p.i., 76.IL10-infected mice had a significantly greater number of splenocytes than MHV-76-infected mice (*P* < 0.0001). Thus, while expression of vIL-10 did not appear to affect the timing of the splenocyte expansion, it did increase its magnitude.

### vIL-10 does not alter the quantity of latent virus in spleen cells or its ability to reactivate

**Figure 6:**
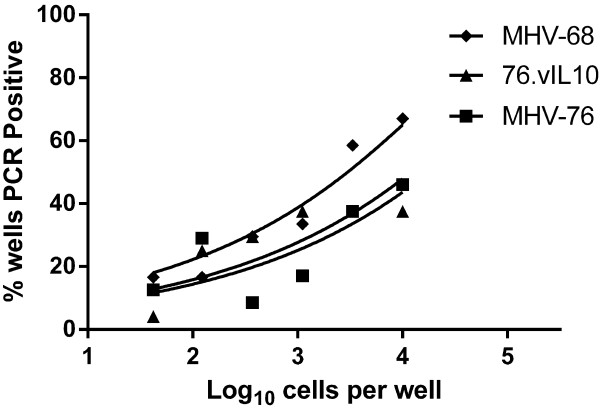
**vIL10 does not affect the quantity of latent virus.** Spleen cells underwent three-fold dilutions with 12 duplicate wells per dilution. Viral DNA was detected by nested PCR to orf50. Curve-fit lines represent results of nonlinear regression analysis. Data points represent the mean of two independent experiments with spleens pooled from five mice per experiment. Results indicate no difference between MHV-76 and recombinants with or without vIL10.

**Figure 7:**
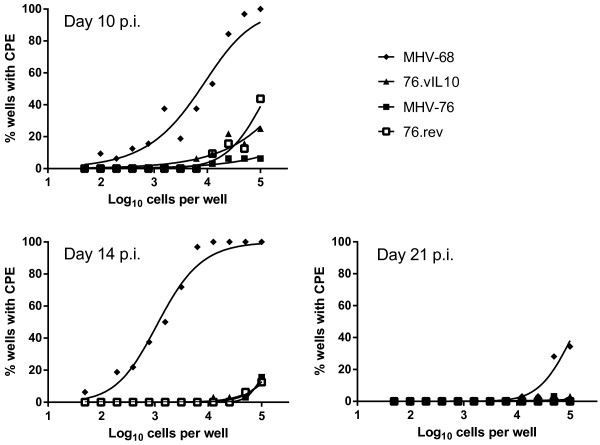
**vIL10 does not affect the quantity of reactivating virus.** Spleen cells were plated atop MEF cells in dilutions across 96-well plates as indicated on the x-axes with 16 duplicate wells per dilution. Wells were scored for CPE on days 10, 14 and 21. Curve-fit lines represent results of nonlinear regression analysis. Data points represent the mean of two independent experiments with spleens pooled from five mice per experiment. Results indicate no difference between MHV-76 and recombinants with or without vIL10.

The presence of viral genomes in splenocytes early in latency was assayed by limiting-dilution PCR. Previous studies have shown a defect in latency for MHV-76 as compared to MHV-68 following i.n. inoculation [44,45]. While Figure 6 does not exhibit a significant difference in PCR-positive cells for the sample size and dilutions tested, MHV-68 trends toward a higher number of positive cells, and MHV-76 and 76.vIL10 track together. Importantly, the expected differences in reactivation between MHV-68 and MHV-76 [44,45] are observed in Figure 7. Data indicate no significant difference in reactivation of MHV-76 with or without vIL-10.

## Discussion

These results provide the first evidence of vIL-10’s role in gammaherpesvirus infection in an *in vivo* model of viral pathogenicity. vIL-10 expression in MHV-76-infected mice increases acute-phase pathogenicity, but does not increase the percentage of latently infected splenocytes or the level of reactivation of latent virus. In accordance with these findings, in EBV, in which vIL-10 is expressed in its native environment, several studies have associated the gene’s expression with the acute phase of infection [13,21,55].

Nevertheless, IL-10 and its ability to drive B cell proliferation and differentiation into plasma cells are considered significant factors in the process of gammaherpesvirus reactivation from latency. The MHV-68 M2 gene product stimulates cIL-10 expression and subsequent B cell proliferation and differentiation [51]. The M2 protein exerts this effect by activating the NFAT signal transduction pathway which induces expression of interferon regulatory factor-4 (IRF-4), in turn inducing cIL-10 expression [52]. cIL-10 expression is also induced in EBV-infected cells by latency-associated LMP-1 and small non-coding RNAs (EBERs) [15,16], and in Kaposi’s sarcoma-associated herpesvirus (KSHV) by viral-encoded miRNA [56]. Whether vIL-10 plays a role in EBV infection in the human similar to that of cIL-10 induction by MHV-68’s M2 protein in the mouse remains to be shown. However, with EBV’s other means of inducing cIL-10 during latency, it is possible that EBV expression of vIL-10 serves a different function. Results presented in this paper suggest that vIL-10’s role may be in enhancing infection during the acute (lytic) phase.

Early expression of vIL-10 homologues by herpesviruses appears to increase the local pool of host cells permissive for infection, thus increasing the chance for trafficking of infected cells to other sites. For example, human cytomegalovirus (HCMV) and RhCMV encode IL-10 homologs that induce the differentiation of macrophages [25], a cell type shown to be permissive for CMV infection [57,58]. Our results have shown that EBV vIL-10 expression by MHV-76 increases splenomegaly, where MHV-induced splenomegaly results, in part, from an increase in B cells [29], and infected B cells are likely vehicles for trafficking MHV-68 from the lung epithelium to the spleen [37].

In addition to increasing the pool of host cells to expand and disseminate primary infection, early expression of viral IL-10 homologues reduces virus-specific effector responses, helping to ensure the survival of infected cells into the latent phase. Such inhibition can occur upstream of effector cell activation by inhibiting innate responses critical to the transition to adaptive immunity. RhCMV IL-10 reduces dendritic cell populations in draining lymph nodes, resulting in a lower frequency of virus-specific T cells [25]. EBV vIL-10 modulates cytokine responses [12], reduces MHC I expression [59], avoids increasing MHC II expression [20], and inhibits monocytes [60]. vIL-10 can also inhibit effector cell responses directly. For example, vIL-10 limits NK cell killing of infected B cells and inhibits CD4+ T cell activity [12]. Studies are planned that will assess effector responses in mice infected with 76.vIL-10.

The timing and level of expression of vIL-10 are likely to relate significantly to vIL-10’s influence on pathogenicity. Expression of vIL-10 by the recombinant virus used in this study was quantified by an ELISA that distinguished vIL-10 from any cIL-10 that might have been produced by host cells. However, it is difficult to know how this level of expression compares to expression of vIL-10 in EBV-infected cells. Published studies have reported vIL-10 concentrations in functional units [14] or fluorescence units [61] rather than in units of mass. Furthermore, the difference in culture conditions for recombinant MHV and EBV would make such comparisons difficult to interpret. Finally, it would be of interest to determine if recombinant viruses package vIL-10 mRNA in the virion and express the product immediately upon infection as has been shown for EBV [12] as well as to ascertain the kinetics of expression of vIL-10 by recombinant MHV in the host animal.

Details are emerging to clarify our understanding of viral modulation of immune responses via cIL-10 and vIL-10. Such understanding may expand our ability to intervene in diseases such as EBV-associated lymphoproliferative disease [62]. Several studies have exploited the immunomodulatory properties of EBV vIL-10 for increasing the survival of allografts [63-70]. The establishment of this model murine gammaherpesvirus expressing vIL-10 may contribute further to such work.

## Conclusions

In this mouse model of gammaherpesvirus infection, EBV vIL-10 appears to influence the acute-phase pathogenicity by increasing the viral titers in lungs and increasing the number of spleen cells, resulting in enhanced splenomegaly. However, following the establishment of latency, vIL-10 expressing strains showed no difference in the percentage of latently infected spleen cells or in the ability of virus to reactivate. Given that EBV and MHV contain other genes that induce cIL-10 expression in latency (e.g. LMP-1 and M2, respectively), vIL-10 may have evolved to serve the specific role in acute infection of enlarging the permissive host cell population, perhaps to facilitate dissemination and initial survival of viral-infected cells.

## Competing interests

The authors declare that they have no competing of interest.

## Authors’ contributions

GL conceived of the study; GL, JPS and JTS designed the study. KG helped generate recombinant herpesviruses and conducted growth curve experiments and animal studies. GL coordinated all and conducted many of the experiments and wrote the manuscript. All authors have approved the final manuscript.
